# New risk factors of postoperative complications in elective gastrointestinal surgery of elderly patients: a prospective cohort study

**DOI:** 10.1186/s12893-021-01171-w

**Published:** 2021-03-30

**Authors:** Kei Yokozuka, Koichi Tomita, Masashi Nakagawa, Itsuki Koganezawa, Shigeto Ochiai, Takahiro Gunji, Yosuke Ozawa, Kosuke Hikita, Toshimichi Kobayashi, Toru Sano, Satoshi Tabuchi, Naokazu Chiba, Eiji Hidaka, Shigeyuki Kawachi

**Affiliations:** Department of Digestive and Transplantation Surgery, Tokyo Medical University Hachioji Medical Center, 1163 Tatemachi, Tokyo, 193-0998 Japan

**Keywords:** Arteriosclerosis, Elderly, Gastrointestinal surgery, Postoperative complication, Skeletal muscle

## Abstract

**Background:**

Gastrointestinal surgery in elderly individuals presents unexpected postoperative complications. However, predicting postoperative complications in elderly patients undergoing gastrointestinal surgeries is challenging because of the lack of a reliable preoperative evaluation system. We aimed to prospectively evaluate three new preoperative assessment methods to predict the postoperative complications in elderly patients undergoing elective gastrointestinal surgery. Moreover, we aimed to identify new risk factors of postoperative complications in this patient group.

**Methods:**

This prospective cohort study enrolled 189 patients (age ≥ 65 years) who underwent elective gastrointestinal surgery at Tokyo Medical University Hachioji Medical Center between April 2017 and March 2019. Assessments performed preoperatively included the biological impedance analysis for evaluating the skeletal muscle mass, the SF-8 questionnaire for evaluating the subjective health-related quality of life, and the blood pressure/pulse wave test for assessing arteriosclerosis. The risk factors for Clavien–Dindo Grade ≥ III postoperative complications were assessed using these new evaluation methods.

**Results:**

Clavien–Dindo Grade ≥ III postoperative complications were observed in 28 patients (14.8%). Univariate and multivariate analyses identified male sex, low skeletal muscle mass, and cardio-ankle vascular index ≥ 10 (arteriosclerosis) as significant independent risk factors of developing Grade ≥ III complications.

**Conclusions:**

Male sex, low skeletal muscle mass, and arteriosclerosis were significant risk factors of postoperative complications in elderly patients undergoing elective gastrointestinal surgery. The obtained knowledge could be useful in identifying high-risk patients who require careful perioperative management.

## Background

The number of elderly people has increased worldwide, especially in Japan. In 2014, approximately 25.9% of the Japanese population were older than 65 years [1]. Furthermore, the average life expectancy of the Japanese population in 2015 was 80.5 years for men and 86.8 years for women, which were the highest worldwide [2].

Elderly patients have a higher incidence of postoperative complications than younger patients. The surgical indication of elective gastrointestinal (GI) surgery for elderly individuals has been determined based on standard preoperative evaluations, such as blood tests, physiological function tests, and performance status. However, unexpected postoperative complications can often occur in elderly patients, unlike in younger patients. Moreover, predicting postoperative complications in elderly patients undergoing gastrointestinal surgeries is challenging because of the lack of a reliable preoperative evaluation system.

This study prospectively evaluated three new preoperative tests to predict the postoperative complications of elective GI surgery in elderly patients. In addition, we aimed to identify new risk factors for developing postoperative complications.

## Methods

This prospective cohort study consecutively enrolled 293 patients who had undergone elective GI surgery at Tokyo Medical University Hachioji Medical Center between April 2017 and March 2019. All patients were aged ≥ 65 years. The procedures were approved by the institutional review board of Tokyo Medical University Hachioji Medical Center (H-164) and performed in accordance with the Helsinki declaration of 1975, as revised in 1983.Written informed consent was obtained from all patients.

In this study, we conducted the following three different evaluations: Biological impedance analysis (BIA) to measure and analyze the body composition; health-related quality of life (QOL) scale to evaluate the subjective mental and physical health; and the blood pressure/pulse wave test to evaluate arteriosclerosis. These evaluations are very easy to perform and require approximately 10 min. They can diagnose low skeletal muscle mass, decreases in QOL, and arteriosclerosis, respectively, which may cause postoperative GI complications. Interestingly, these conditions could not be evaluated by conventional preoperative examinations for GI surgery.

**Fig. 1:**
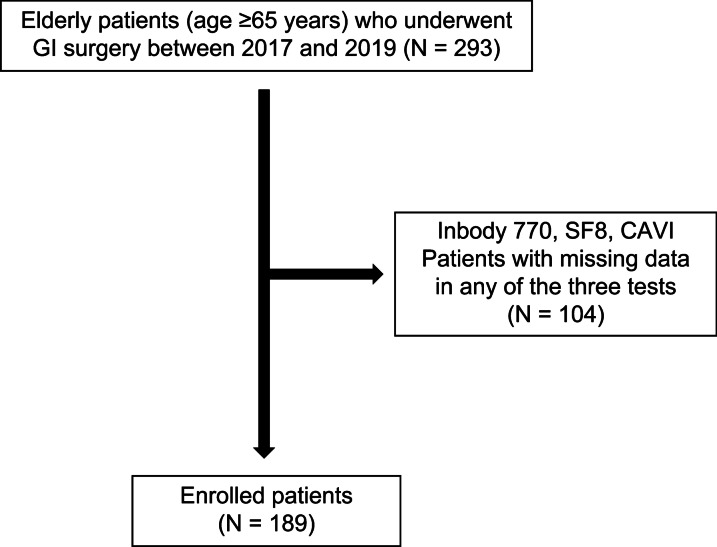
Study flowchart. Of the 293 patients, 104 were excluded because of missing data in any of the three evaluations. Finally, 189 patients were included in this study. *GI* gastrointestinal, *CAVI* cardio-ankle vascular index

A total of 104 patients were excluded from this study because of missing data in any of the three evaluations. For BIA, patients with metal objects internally, such as a cardiac pacemaker or those who could not stand independently, were excluded. For the health-related QOL scale, those who could not respond to the questionnaires were excluded. Regarding the blood pressure pulse wave tests, patients in whom the limb blood pressure could not be measured because of a history of hemodialysis or breast cancer surgery or because of their inability to extend their limbs were excluded. Finally, 189 patients were enrolled in this study (Fig. 1). The 5-item modified frailty index (5-mFI) was also calculated for all patients to compare these three evaluations. The 5-mFI is a valid predictor of postoperative outcomes, consisting of diabetes mellitus, congestive heart failure, hypertension, chronic obstructive pulmonary disease, and functional health status [3, 4].

### Biological impedance analysis

The multifrequency body composition analyzer InBody 770 (Biospace Co., Seoul, Korea) was used. This device can measure body water, muscle, and fat using the impedance method. In this study, the skeletal muscle weight, skeletal muscle index (SMI), body fat (kg), and body cell mass (kg) were measured. SMI values < 7.0 kg/m^2^ for men and < 5.7 kg/m^2^ for women were considered abnormal, based on the criteria of the Asian Working Group on Sarcopenia [5].

### Health‐related QOL scale by SF-8

The health-related QOL scale was investigated using the Medical Outcomes Study 8-item Short-Form Health Survey (SF-8™) questionnaire [6]. The SF-8 is a measure of comprehensive health-related QOL. SF-8 measures the following two components: the physical and the mental component summary. The cutoff value of SF-8 was defined as < 25th percentile of the normal distribution, and patients with lower scores than the cutoff value were diagnosed as having abnormal subjective QOL.

### Blood pressure/pulse wave test

The cardio-ankle vascular index (CAVI) was measured using the blood pressure pulse wave analyzer VaSera VS-2000 (Fukuda Denshi Tokyo Nishi Sales Co., Ltd., Tokyo, Japan) [7]. In general, the CAVI is measured by placing a heart sound microphone around the cardiac apex and a manchette around the limbs, and it is calculated using an algorithm [8]. The CAVI is considered a good index of arteriosclerosis [9] and is positively correlated with arteriosclerosis parameters [10]. There is no standard cutoff CAVI value in patients who had undergone GI surgery. In this study, CAVI scores ≥ 10.0 were considered abnormal (arteriosclerosis) based on the preliminary study (data not shown). As no apparent sex difference was reported for the CAVI values, the analysis was not stratified by sex.

### Postoperative complications

The severity of postoperative complications was graded using the Clavien–Dindo classification [11, 12]. This is a representative grading system of postoperative complications used worldwide. We aimed to predict the complications of Grade ≥ III, because they require invasive treatments. Pulmonary complications include pneumonia, atelectasis, and hypoxia requiring reintubation.

### Statistical analyses

All statistical analyses were performed using IBM SPSS Statistics for Windows, version 26.0, (IBM Corp., Armonk, NY, USA). Continuous variables are expressed as means ± standard deviations or medians with ranges. The Mann–Whitney U-test was used for their comparisons. Categorical variables were compared using the Chi-square or Fisher’s exact test, as required. Throughout our analysis, statistical tests were two-tailed, and the significance was set at p < 0.05. A multivariate regression analysis was performed using a logistic regression analysis with forward likelihood ratio selection.

## Results

### Patient characteristics

The patient characteristics are summarized in Table 1. Esophageal surgery, gastrectomy, gastric bypass, colectomy, rectectomy, stoma closure, and appendectomy were performed in two, 67, seven, 77, 27, nine, and three cases, respectively. Among our patients, 23 (12.1%), 21 (11.1%), and six (3.0%) had Clavien–Dindo Grade II (requiring drug therapy, such as antibiotics), III (requiring invasive therapy), and IV complications (involving organ dysfunction and requiring admission to the intensive care unit), respectively. A Grade V complication (death) occurred in one patient (0.5%). Twenty-eight patients (14.8%) had Grade ≥ III complications, including abdominal abscess, pulmonary complications, ileus, pancreatic fistula, and anastomotic leakage in nine, seven, six, six, and three patients, respectively. Some patients had multiple complications.

**Table 1:** Characteristics of the study population

	All cases	Upper GI	Lower GI
	N = 189*	N = 76	N = 119
General background			
Age (years)	75 (65–95)	75 (65–92)	75 (65–95)
Sex			
Male	125 (66.1%)	54 (71.0%)	72 (62.0%)
Female	64 (33.8%)	22 (28.9%)	44 (37.9%)
Comorbidity			
Hypertension	100 (52.9%)	42 (55.2%)	60 (51.7%)
Diabetes mellitus	47 (24.8%)	16 (21.0%)	30 (25.8%)
Hyperlipidemia	34 (17.9%)	16 (21.0%)	17 (14.6%)
Cardiovascular	26 (13.7%)	11 (14.4%)	16 (13.7%)
Cerebrovascular	18 (9.5%)	5 (6.5%)	12 (10.3%)
Renal	11 (5.8%)	3 (3.9%)	8 (6.8%)
Hemodialysis	5 (2.6%)	0 (0.0%)	5 (4.3%)
Respiratory	4 (2.1%)	3 (3.9%)	1 (0.8%)
5-item modified frailty index	0.98 ± 0.889	0.99 ± 0.893	0.97 ± 0.864
Surgical information			
Organ			
Esophagectomy		2 (2.6%)	
Gastrectomy		67 (88.2%)	
Distal		37 (48.6%)	
Total		14 (18.4%)	
Proximal		8 (10.5%)	
Partial		8 (10.5%)	
Bypass		7 (9.2%)	
Colectomy			77 (64.7%)
Rectectomy			27 (22.7%)
Small intestine			1 (0.8%)
Stoma closure			9 (7.7%)
Ileostomy/colostomy			2 (1.7%)
Appendectomy			3 (2.5%)
Disease			
Malignant	168 (88.8%)	68 (89.4%)	106 (91.3%)
Benign	21 (11.1%)	8 (10.5%)	10 (8.6%)
Approach			
Open laparotomy	78 (41.2%)	53 (69.7%)	33 (28.4%)
Laparoscopic	111 (58.7%)	23 (30.2%)	83 (71.5%)
New preoperative evaluation			
InBody 770			
BMI (kg/m²)	22.5 (14.6–37.6)	21.9 (14.6–32.8)	22.8 (15.9–37.6)
SMI (kg/m²)			
Men	7.1 (4.1–10.2)	7.0 (4.1–10.2)	7.10 (5.20–9.20)
Women	5.8 (4.2–10.4)	5.8 (4.8–7.8)	5.8 (4.2–10.4)
Abnormal	78 (41.2%)	35 (46.0%)	45 (38.7%)
Body fat (%)	26.0 (7.1–47.0)	24.5 (7.1–43.9)	26.4 (12.1–47.0)
Body cell mass (kg)	26.7 (16.8–42.2)	26.7(16.8–42.2)	26.9 (17.5–38.2)
SF-8			
PCS score			
Men	47.4 (18.6–59.2)	48.0 (19.2–57.5)	46.4 (18.6–59.2)
Women	48.4 (19.6–58.8)	47.3 (19.6–57.5)	49.0 (23.6–58.8)
Abnormal	43 (22.7%)	13 (17.1%)	31 (26.7%)
MCS score			
Men	49.3 (19.9–64.6)	50.4 (26.6–64.6)	48.9 (19.9–64.6)
Women	48.0 (24.4–57.3)	48.6 (29.7–55.3)	46.5 (24.4–57.3)
Abnormal	74 (39.1%)	27 (35.5%)	49 (42.2%)
Blood pressure/pulse wave test			
CAVI	9.31 ± 1.862	9.23 ± 1.879	9.36 ± 1.856
Abnormal (≥10)	63 (33.3%)	24 (31.5%)	41 (35.3%)
Surgical outcome			
Operative time (min)	244.0 (65–788)	261.5 (100–587)	217.0 (65–788)
Blood loss (g)	40.0 (10–2995)	66.5 (10–1635)	20.0 (10–2995)
Postoperative complication			
Clavien–Dindo Grade			
I	6 (3.1%)	2 (2.6%)	4 (3.4%)
II	23 (12.1%)	12 (15.7%)	11 (9.4%)
III	21 (11.1%)	10 (13.1%)	12 (10.3%)
IV	6 (3.2%)	3 (3.9%)	3 (2.5%)
V	1 (0.5%)	0 (0.0%)	1 (0.9%)
≥ III	28 (14.8%)	13 (17.1%)	16 (13.7%)
Type of complication (Grade ≥ III)			
Abdominal abscess	9 (4.7%)	5 (6.5%)	4 (3.4%)
Pulmonary complications	7 (3.7%)	5 (6.5%)	2 (1.7%)
Ileus	6 (3.1%)	0 (0.0%)	6 (5.1%)
Pancreatic fistula	6 (3.1%)	5 (6.5%)	1 (0.9%)
Anastomotic leakage	3 (1.5%)	2 (2.6%)	1 (0.9%)
Anastomotic bleeding	1 (0.5%)	0 (0.0%)	1 (0.9%)
Anastomotic stenosis	1 (0.5%)	1 (1.3%)	0 (0.0%)
Cerebral infarction	1 (0.5%)	1 (1.3%)	0 (0.0%)
Intestinal ischemia	1 (0.5%)	0 (0.0%)	1 (0.9%)
Myocardial infarction	1 (0.5%)	0 (0.0%)	1 (0.9%)
Postoperative stay (days)	12 (3–197)	13 (6–93)	11 (3–197)

Continuous variables are expressed as mean values ± standard deviations or medians with ranges

Categorical variables are expressed as number of patients

*BMI* body mass index, *SMI* skeletal muscle mass index, *PCS* physical component summary, *MCS* mental component summary, *ABI*, ankle-brachial pressure index, *CAVI* cardio-ankle vascular index, *GI* gastrointestinal

^a^Six cases were duplications of upper and lower GI

### Univariate and multivariate analyses

The results of the univariate and multivariate analyses evaluating the relationship between the parameters obtained using the new evaluation techniques and Grade ≥ III complications are presented in Table 2. In the univariate analysis, there was a significant association of Grade ≥ III complications with male sex (p = 0.005), hypertension (p = 0.033), abnormal SMI (p = 0.007), and CAVI ≥ 10 (p = 0.004). Using these factors in the multivariate logistic regression analysis, male sex (odds ratio [OR], 5.51; 95% confidence interval [CI] 1.55–19.57; p = 0.008), abnormal SMI (OR, 2.78; 95% CI 1.16–6.71; p = 0.023), and CAVI ≥ 10 (OR, 2.81; 95% CI 1.18–6.67; p = 0.019) remained independent risk factors of postoperative complications. The abnormal SMI indicated low skeletal muscle mass, and CAVI ≥ 10 indicated arteriosclerosis. The 5-item modified frailty index did not show a significant difference for Grade ≥ III postoperative complications in these subjects.

**Table 2:** Analyses of clinical characteristics of patients with postoperative complications assessed using the Clavien–Dindo classification

	Univariate analysis				Multivariate analysis	
	Grade ≤ II	Grade ≥ III	p-value	Odds ratio (95% CI)	p-value	Odds ratio (95% CI)
	N = 161	N = 28				
General background						
Age						
Years	75.0 (65–95)	76.5 (67–92)	0.206			
≥ 80 years	36 (22.4%)	11 (39.3%)	0.056	2.25 (0.97–5.23)	0.088	
Sex						
Male	100 (62.1%)	25 (89.3%)	0.005	5.08 (1.47–17.55)	0.008	5.51 (1.55–19.57)
Comorbidity						
Hypertension	80 (49.7%)	20 (71.4%)	0.033	2.53 (1.05–6.08)	0.094	
Diabetes mellitus	41 (25.5%)	6 (21.4%)	0.648			
Hyperlipidemia	29 (18.0%)	5 (17.9%)	0.984			
Cardiovascular	22 (13.7%)	4 (14.3%)	0.563			
Cerebrovascular	14 (8.7%)	4 (14.3%)	0.266			
Renal	8 (5.0%)	3 (10.7%)	0.211			
Hemodialysis	4 (2.5%)	1 (3.6%)	0.556			
Respiratory	2 (1.2%)	2 (7.1%)	0.105			
5-item modified frailty index						
≥ 2	35 (21.9%)	10 (35.7%)	0.113			
Disease						
Malignant	143 (88.8%)	25 (89.3%)	0.621			
InBody 770						
BMI (kg/m^2^)	22.5 (14.6–37.6)	21.9 (15.7–26.5)	0.215			
SMI	6.6 (4.1–10.4)	6.55 (5.3–8.3)	0.538			
Abnormal	60 (37.3%)	18 (64.3%)	0.007	3.03 (1.31–6.99)	0.023	2.78 (1.16–6.71)
Body fat (%)	26.3 (7.1–47.0)	25.1 (9.0–40.0)	0.569			
Body cell mass (kg)	26.8 (16.8–42.2)	26.6 (22.1–34.3)	0.871			
SF-8						
PCS score	47.9 (18.6–59.2)	46.5 (19.2–55.8)	0.295			
Abnormal	34 (23.4%)	9 (36.0%)	0.182			
MCS score	48.8 (24.4–64.6)	51.6 (20.0–64.7)	0.291			
Abnormal	65 (44.8%)	9 (36.0%)	0.411			
Blood pressure/pulse wave test						
CAVI						
Abnormal	47 (29.2%)	16 (57.1%)	0.004	3.23 (1.42–7.36)	0.019	2.81 (1.18–6.67)

Continuous variables are expressed as mean values ± standard deviations or medians with ranges

Categorical variables are expressed as number of patients

*BMI* body mass index, *SMI* skeletal muscle mass index, *PCS* physical component summary, *MCS* mental component summary, *CAVI* cardio-ankle vascular index, *CI* confidence interval

## Discussion

The risk factor of postoperative complications in elective GI surgery, especially of elderly patients, has not been well established. For example, the body mass index or laparoscopic conversion, which has been considered the risk factors, were not proved as the risk factors of postoperative complications based on the previous reports [13, 14]. According to the recent report of Geriatric Oncology Surgical Assessment and Functional rEcovery after Surgery (GOSAFE) study, which included 997 multinational patients, the frailty is frequent in older patients undergoing cancer surgery [15]. The present study evaluated three new preoperative tests for predicting postoperative complications in elderly patients who had undergone elective GI surgery, and male sex, low skeletal muscle mass, and arteriosclerosis were found to be independent risk factors of postoperative complication.

Although the study included elderly patients aged ≥ 65 years, age did not significantly impact their condition. Previous systematic reviews and literature findings indicated that age might not be significantly associated with postoperative complications [16], which was consistent with our findings. A possible explanation may be that surgery or preoperative management in this study would be performed more attentively in older patients.

In our study, male sex was found to be a significant risk factor for developing postoperative complications. Moreover, women had more prominent hormonal and cell-mediated immune responses compared to men [17]. Conversely, men had higher testosterone and lower estradiol levels during surgery and other interventions than women, which may be a factor involved in immunosuppression and may contribute to increased risk for postoperative complications [18].

In elderly patients, the combination of age-related decrease in muscle mass (primary sarcopenia) and disease-related loss of activity or muscle loss because of malnutrition (secondary sarcopenia) has been reported to be a factor that affects the prognosis of various GI surgical interventions, such as living donor liver transplantation [19], hepatectomy [20], and pancreatectomy [21]. In BIA, body fat mass was also reported to predict postoperative complications [22], as body fat makes surgery difficult and increases the amount of blood loss, surgical time, and wound infection risk. Nonetheless, only SMI was found to be a significant risk factor in our study.

Concerning the health-related QOL scale, assessing a patient’s preoperative status using SF-36 may have a greater value, as the preoperative status could be an independent risk factor of postoperative complications in patients undergoing major GI surgeries, as suggested by a previous report [23]. As the mental and physical health of the patients is largely perceived by them, there may have been a discrepancy between the patients’ actual condition and their answers to the self-reported questionnaire.

To our knowledge, there is currently no study directly elucidating the relationship between arteriosclerosis and GI surgical complications. The CAVI, which was newly introduced in the present study, is an index of arteriosclerosis in blood pressure/pulse wave test. Interestingly, the ankle-brachial pressure index is an index of peripheral arteriosclerosis, whereas the CAVI is an index of visceral arteriosclerosis. Our results showed that patients with diabetes mellitus or chronic kidney disease had higher rates of abnormal CAVIs, indicating higher arteriosclerosis rates. The visceral arteriosclerosis might be related to some postoperative complications after GI surgery, such as ileus, pancreatic fistula, anastomotic leakage, bleeding, stenosis, cerebral infarction, intestinal ischemia, and myocardial infarction. Moreover, CAVI was previously reported to be an independent risk factor for frailty [24], which may cause additional complications.

There were several limitations in this study. First, this was a single-institutional study with limited sample size; thus, a multi-institutional study may be needed to confirm our results. Moreover, patients with poor general conditions (inability to stand or answer the questionnaire) could not be evaluated using the assessment tools used in this study; thus, other evaluation methods will be needed for them.

## Conclusions

In elderly patients undergoing elective GI surgery, male sex, low skeletal muscle mass, and arteriosclerosis were significant risk factors of presenting postoperative complications. The knowledge of these risk factors could be useful in identifying high-risk patients requiring careful perioperative management.

## Data Availability

All data generated or analyzed during this study are included in this published article.

## Abbreviations

GI: Gastrointestinal
BIA: Biological impedance analysis
SMI: Skeletal muscle index
QOL: Health-related quality of life
CAVI: Cardio-ankle vascular index
